# Supplementary material for: A Long-Term Incidence of Heart Failure and Predictors Following Newly Developed Acute Myocardial Infarction: A 10 Years Retrospective Cohort Study with Korean National Health Insurance Data
Source: Int J Environ Res Public Health. 2021 Jun 8;18(12):6207. doi: 10.3390/ijerph18126207 (PMC8229614; doi:10.3390/ijerph18126207)
Supplement: Supplementary file 1 [file ijerph-18-06207-s001.zip › ijerph-1227393-supplementary.pdf]

Supplemental Material

Table S1. Demographic characteristics and pre-existing conditions of newly developed AMI patients in 2010.

	**Total**		**Men**		**Women**		**p–Value**
	**(n = 18,328)**		**(n = 12,403)**		**(n = 5,929)**		
	**N**	**(%)**	**N**	**(%)**	**N**	**(%)**	
Age							
Mean ± SD	66.0 ± 12.4		62.5 ± 11.8		73.4 ± 10.4		
40–49	2059	(11.2)	1921	(15.5)	138	(2.3)	
50–59	3918	(21.4)	3432	(27.7)	486	(8.2)	
60–69	4547	(24.8)	3299	(26.6)	1248	(21.0)	<0.0001
70–79	5066	(27.6)	2765	(22.3)	2301	(38.8)	
80+	2738	(14.9)	986	(7.9)	1752	(29.5)	
Type of insurance							
NHI	16,706	(91.2)	11,660	(94.0)	5046	(85.1)	<0.0001
Medical Aid	1622	(8.8)	743	(6.0)	879	(14.8)	
Type of AMI							
STEMI	2507	(13.7)	1636	(13.2)	871	(14.7)	
NSTEMI	4985	(27.2)	3560	(28.7)	1425	(24.1)	<0.0001
Unspecified	10,836	(59.1)	7207	(58.1)	3629	(61.2)	
Severity							
Moderate	14,809	(80.8)	10,414	(84.0)	4395	(74.1)	<0.0001
Severe	3519	(19.2)	1989	(16.0)	1530	(25.8)	
Pre–existing conditions							
Hypertension							
No	6718	(36.7)	5441	(43.9)	1277	(21.5)	<0.0001
Yes	11,610	(63.3)	6962	(56.1)	4648	(78.4)	
Diabetes							
No	11,797	(64.4)	8423	(67.9)	3374	(56.9)	<0.0001
Yes	6531	(35.6)	3980	(32.1)	2551	(43.1)	
Dyslipidemia							
No	12,873	(70.2)	8883	(71.6)	3990	(67.3)	<0.0001
Yes	5455	(29.8)	3520	(28.4)	1935	(32.6)	

N: number, SD: standard deviation, NHI: National Health Insurance, STEMI: ST segment Elevation Myocardial Infarction, NSTEMI: non-ST segment Elevation Myocardial Infarction. P value for Chi-square test.
